# Enhanced Interfacial Plasma Degradation of Per- and Polyfluoroalkyl Substances (PFAS) via Ultrasonically Generated Microdroplets

**DOI:** 10.3390/molecules31071157

**Published:** 2026-03-31

**Authors:** Ao Chen, Haoyu Yuan, Zhengtong Qiu, Chaonan Mu

**Affiliations:** State Key Laboratory of Marine Resource Utilization in South China Sea, School of Marine Sciences, Hainan University, Haikou 570228, China; ca1508686802@163.com (A.C.);

**Keywords:** PFAS, microdroplet interfaces, ultrasonic atomization, non-thermal plasma, interfacial degradation

## Abstract

The exceptional stability of C-F bonds renders PFAS highly persistent in aqueous environments, posing significant challenges for conventional treatment technologies. While plasma-based technologies show promise, their efficiency is often limited by poor gas–liquid mass transfer in bulk liquid. Here, an in-house constructed ultrasonic atomization–dielectric barrier discharge (UEN-DBD) system was developed to promote PFAS degradation under non-thermal plasma conditions. Ultrasonic atomization generated microdroplets, which promoted PFAS enrichment at the surface of microdroplets and facilitate interactions with plasma-generated reactive species. Using perfluorooctanoic acid (PFOA) and perfluorooctanesulfonate (PFOS) as model compounds, degradation behavior was evaluated over an initial concentration range of 0.01–1.0 ppm. At 0.01 ppm, degradation efficiencies of 96.06% for PFOA and 94.86% for PFOS were achieved within 5 min. Electron paramagnetic resonance (EPR) spectroscopy confirmed the formation of oxidative radicals (·OH) and suggested a mixed redox environment involving reactive species, potentially including superoxide (O_2_·^−^) or hydrated electrons (e_aq_^−^), in the discharge-treated system. High-resolution mass spectrometry results are consistent with a stepwise chain-shortening pathway dominated by successive –CF_2_– scission, while fluoride-release measurements provided supporting evidence for partial defluorination. These findings advance the understanding of plasma-assisted PFAS degradation at the gas–liquid interface and provide a basis for the further development of plasma-assisted PFAS treatment strategies.

## 1. Introduction

Per- and polyfluoroalkyl substances (PFAS) are a broad class of synthetic organofluorine compounds extensively utilized in firefighting foams, nonstick coatings, and water-repellent textiles, primarily due to their high thermal stability and unique amphiphobic properties [1]. The strong C-F bonds in PFAS compounds confer exceptional resistance to thermal, chemical, and biological degradation, leading to their ubiquitous distribution, long-term persistence, and consequently raising concerns regarding ecological and human health risks [2,3]. Perfluorooctanoic acid (PFOA) and perfluorooctane sulfonate (PFOS) are among the most extensively studied PFAS and are regulated as persistent organic pollutants (POPs) under the Stockholm Convention. Reported half-lives for these compounds in aquatic environments reach up to 41 and 92 years, respectively [4]. Their extreme persistence and chemical stability render the effective treatment of PFAS-contaminated water a significant challenge.

Conventional treatment methods, including adsorption, ion exchange, and membrane separation, can effectively remove PFAS from water but do not break down their chemical structure [5]. Instead, PFAS remain intact and are concentrated in waste streams, requiring further treatment and may pose additional risks [6,7]. Advanced oxidation processes (AOPs) have been explored using in situ generated highly reactive oxygen species (e.g., ·OH, O_3_, H_2_O_2_) to initiate chemical degradation of persistent contaminants, yet their effectiveness remains limited for these persistent compounds [8,9,10]. Dielectric barrier discharge (DBD) plasma has attracted increasing attention as a non-thermal technique capable of facilitating redox reactions for PFAS degradation under ambient conditions [11,12,13]. When applied to bulk aqueous systems, DBD plasma often suffers from limited interfacial contact between reactive species and target molecules, leading to inefficient utilization of these species [13,14]. This drawback, combined with the short lifetime of reactive species and diffusion limitations in bulk liquid, makes it difficult to achieve efficient PFAS degradation in conventional plasma reactors. To improve gas–liquid mass transfer, ultrasonic atomization and cavitation create fine droplets and bubbles, which increase interfacial area and reduce diffusion distances [15]. Interfacial microdroplets can facilitate C–F bond activation even without external energy input, as evidenced by the spontaneous PFOA degradation observed at microdroplet interfaces [16]. Zhang’s group reported an order-of-magnitude enhancement in the photochemical decomposition of PFOA at the microdroplet surfaces compared to the bulk phase. This was attributed to the synergistic effects of interfacial enrichment, ultrahigh interfacial electric fields, and partial solvation at the interface [17]. Furthermore, microdroplet interfacial effects have been shown to promote O_2_^−^-mediated nucleophilic reactions, enabling complete PFOA destruction with high defluorination efficiency [18]. Engineering the interfacial microenvironment thus represents a promising strategy for PFAS degradation. While ultrasonic atomization offers a practical means to generate micron-sized droplets and introduce cavitation-related effects, non-thermal plasma provides reactive oxygen species and electrons necessary for C-F bond activation. How microdroplet-mediated interfacial enrichment influences plasma-assisted degradation efficiency remains insufficiently understood [9,10,11,12].

Herein, a coupled ultrasonic atomization–dielectric barrier discharge (UEN-DBD) system was developed to investigate the plasma-assisted degradation of PFAS at the gas–liquid interface. Using PFOA and PFOS as model contaminants, the enrichment behavior driven by nebulization and the resulting degradation efficiency were quantitatively evaluated via liquid chromatography–mass spectrometry (LC-MS). The effects of initial concentration and treatment time on reaction kinetics were systematically examined to elucidate the relationship between interfacial enrichment and energy utilization. Electron paramagnetic resonance (EPR) spectroscopy and high-resolution mass spectrometry were employed to provide qualitative information on the reactive species and to infer possible degradation intermediates. These observations offer insights into interfacial plasma chemistry and may help guide the development of technologies for treating persistent contaminants.

## 2. Results and Discussion

### 2.1. Enrichment and Degradation Behavior of PFASs in the UEN–DBD System

**Figure 1:**
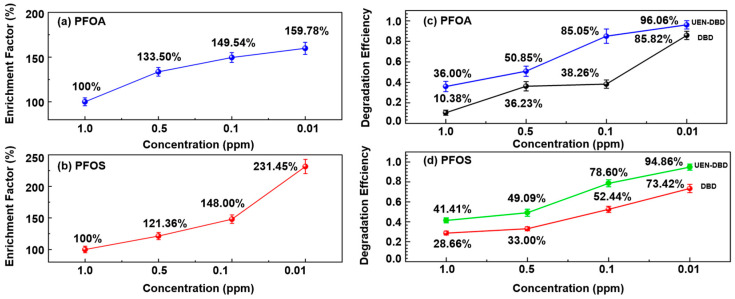
Enrichment factors and degradation efficiencies of PFOA (**a**,**c**) and PFOS (**b**,**d**) as a function of initial concentration under DBD and UEN–DBD treatment.

PFAS concentrations before and after ultrasonic atomization were quantified by LC-MS to evaluate interfacial enrichment behavior. Here, the ‘after atomization’ concentration refers to the condensate collected from atomized microdroplets, and the enrichment factor is defined as C_after_/C_0_; details are provided in the Supporting Information. As shown in Figure 1a,b, both PFOA and PFOS followed concentration-dependent enrichment behavior over the investigated concentration range (1.0–0.01 ppm). At an initial concentration of 1.0 ppm, the enrichment factor remained near 100%, indicating that the microdroplet interface was likely saturated or that the bulk-to-surface partition was limited by the relatively high solute-to-solvent ratio. As the initial concentration decreased to 0.01 ppm, the enrichment factor reached 159.78% for PFOA and a substantial 231.45% for PFOS (Table 1). This trend aligns with the Gibbs adsorption isotherm, where molecules with higher surface activity (such as the longer-chain PFOS) exhibit stronger spontaneous accumulation at the gas–liquid interface as the bulk concentration approaches the trace range.

The interfacial partitioning of PFAS directly governs the degradation kinetics within the plasma–microdroplet microenvironment. As illustrated in Figure 1c,d, the degradation efficiencies under different treatment conditions were further evaluated. The detailed degradation data are presented in Table 2. For both PFOS and PFOA, degradation efficiencies increased as the initial concentration decreased. Across the entire concentration range (0.01–1.0 ppm), the UEN-DBD system exhibited higher degradation efficiencies than the standalone DBD process. For PFOA, degradation efficiencies in the DBD process increased from 10.38% at 1.0 ppm to 85.82% at 0.01 ppm, whereas the corresponding values for the UEN-DBD system increased from 36.00% to 96.06%. Notably, at an intermediate concentration of 0.1 ppm, UEN-DBD achieved a degradation efficiency of 85.05%, more than twice that obtained by DBD alone (38.26%). Such enhancement likely stems from the improved mass transfer of short-lived reactive species in the UEN-DBD system [17,18]. In conventional DBD systems, the reaction is restricted by the diffusion of pollutant from the bulk to surface and the limited penetration depth of reactive species. Ultrasonic atomization delivers PFAS-enriched microdroplets directly into the discharge region, ensuring the immediate encounter between interfacial PFAS and short-lived reactive species.

**Figure 2:**
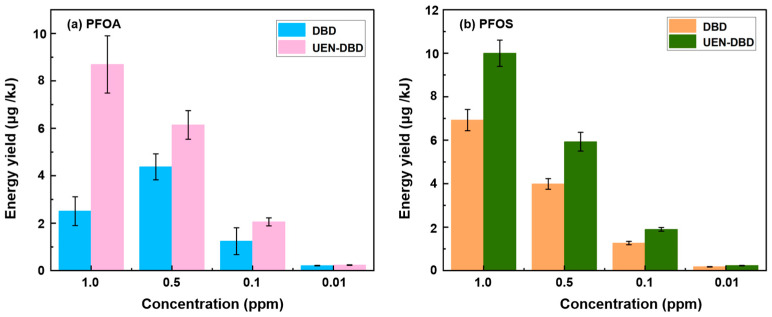
Comparison of energy yield for PFOA and PFOS degradation in standalone DBD and UEN–DBD systems at varying initial concentrations.

To evaluate the energy utilization efficiency of the DBD and UEN–DBD systems, the energy yield was calculated as the mass of PFAS removed per unit electrical energy input (μg kJ^−1^). Representative steady-state voltage and current waveforms applied to the DBD reactor are provided in the Supporting Information (Figure S4). The detailed definition, calculation procedure, and energy-accounting boundary are provided in the Supporting Information. As shown in Figure 2 and Table 3, both PFOA and PFOS exhibited a strong dependence on the initial concentration. In the standalone DBD treatment, the energy yield of PFOA reached a maximum at 0.5 ppm (~4.38 μg kJ^−1^) and followed by a slight decrease at 1.0 ppm, indicating an optimal pollutant loading for reactive species utilization. At low concentrations, the limited availability of target molecules leads to inefficient consumption of reactive species. At higher concentrations, interfacial saturation and competition from intermediate products may partially restrict further energy utilization. In contrast, the UEN–DBD system maintained consistently higher energy yields across all tested concentration ranges. For both PFOA and PFOS, the yields increased progressively with initial concentration up to 1.0 ppm, reaching maximum values of ~8.70 and 10.00 μg kJ^−1^, respectively. The absence of a yield plateau in the UEN-DBD system confirms that the continuous generation of microdroplets effectively refreshes the reactive interface and bypasses the saturation limits inherent in bulk-liquid reactors. Notably, PFOS generally exhibited higher energy yields than PFOA under identical conditions, which is consistent with its stronger interfacial activity and higher enrichment factors (Figure 1a,b). While the absolute energy yields in this 50 mL laboratory system remain lower than those reported for large-volume DBD industrial reactors operated at higher PFAS concentrations, the significant relative enhancement confirms that engineering the microdroplet–plasma interface is a robust strategy for improving the energy utilization of PFAS remediation.

**Figure 3:**
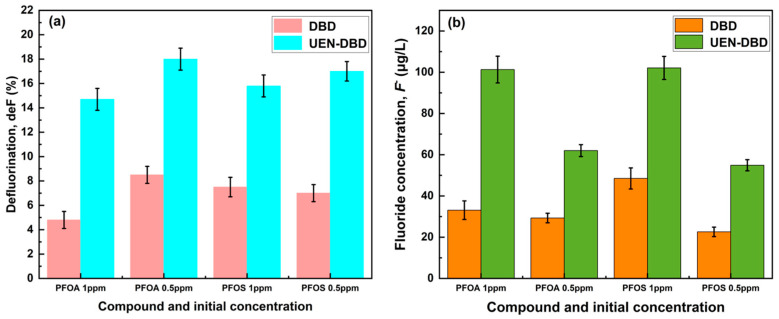
Defluorination and fluoride release of PFOA and PFOS after 5 min treatment (1.0 and 0.5 ppm). (**a**) Defluorination (deF, %); (**b**) fluoride concentration in the collected condensate (*F*^−^, µg/L).

To further assess whether the observed parent-PFAS removal is accompanied by C–F bond cleavage, fluoride ions (*F*^−^) released into the collected condensate were quantified for representative PFOA and PFOS solutions at initial concentrations of 1.0 and 0.5 ppm, and the defluorination degree (deF, %) was calculated from fluoride release relative to the initial total fluorine of the parent PFAS (PFOA: *n_F_* = 15; PFOS: *n_F_* = 17; calculation details are provided in the Supporting Information). As summarized in Figure 3 and Table 4, measurable *F*^−^ release was detected after 5 min treatment for both PFOA and PFOS. Compared with standalone DBD, the UEN-DBD configuration consistently yielded higher *F^−^* concentrations and deF values under the same operating conditions, suggesting enhanced defluorination in the microdroplet–plasma environment. It is also noted that the deF values remain lower than the parent-compound removal, implying that a fraction of fluorine is likely retained in transformation products rather than being fully converted to inorganic fluoride within the present treatment time. Overall, these fluoride-release results provide direct support for partial defluorination during UEN-DBD treatment and strengthen the interpretation of interface-enhanced PFAS removal.

**Figure 4:**
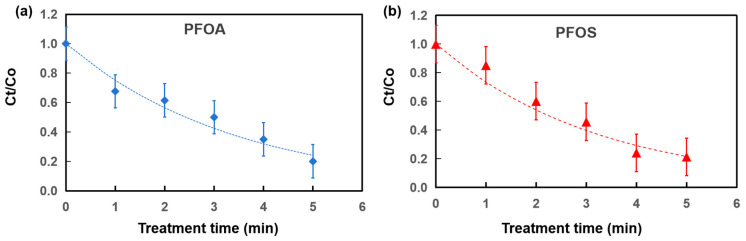
Time-dependent degradation in the UEN-DBD system at an initial concentration of 0.1 ppm: (**a**) PFOA; (**b**) PFOS.

Time-resolved degradation experiments were performed at an initial concentration of 0.1 ppm to further elucidate the influence of interfacial enrichment on reaction rates. As illustrated in Table 5 and Figure 4, the normalized concentration (C_t_/C_0_) for both PFOA and PFOS displayed a progressive exponential decay with increasing treatment time. The degradation profiles were well-fitted by a pseudo-first-order kinetics (detailed calculation parameters are provided in the Supporting Information), consistent with previous studies on PFAS degradation in non-thermal plasma systems [19]. The apparent rate constants (*k*) for PFOS (0.307 min^−1^) were slightly higher than that of PFOA (0.288 min^−1^), which corroborates the superior interfacial enrichment of sulfonated PFAS. Despite its higher kinetic constant, PFOS showed a slightly higher residual concentration (21.4 ppb) than PFOA (20.0 ppb) after 5 min of treatment, which is attributed to its higher initial interfacial loading. Owing to its stronger hydrophobicity, a larger fraction of PFOS partitions into the microdroplet boundary layer during atomization, resulting in a higher local molecular density within the discharge region. Under these conditions, the UEN–DBD system sustains efficient degradation by exploiting the high reaction activity at the microdroplet interface, even at elevated interfacial loadings.

### 2.2. Identification of Reactive Species

**Figure 5:**
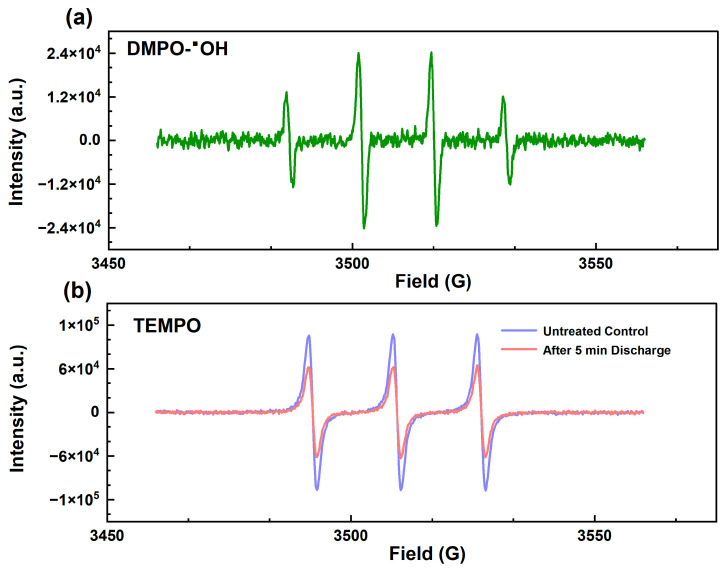
Identification of plasma-generated reactive species via EPR spectroscopy. (**a**) Detection of ·OH radicals using DMPO as a spin trap; (**b**) TEMPO quenching as an indicator of reducing/active species generated during discharge.

Electron paramagnetic resonance (EPR) spectroscopy was employed to identify the reactive species generated in the UEN-DBD system. As depicted in Figure 5, a typical quartet signal with an intensity ratio of 1:2:2:1 was detected in the presence of DMPO spin trap after plasma discharge. This signal was assigned to the DMPO-OH adduct, confirming abundant ·OH formation. The appearance of this signal, which was absent in the control experiment, indicates that ·OH is a representative short-lived reactive species produced during the discharge process. Since ·OH possesses a high oxidation potential (2.80 V) [20], it plays a pivotal role in attacking the C-F bonds of PFAS. In addition, TEMPO was used as a spin probe to qualitatively assess the presence of reducing/redox-active species in the UEN-DBD system (Figure 5). TEMPO is a stable nitroxide radical with a well-defined three-line EPR signal. In the presence of strong reducing species (including e_aq_^−^), TEMPO could be reduced to its diamagnetic hydroxylamine form (TEMPO-H), resulting in attenuation or disappearance of the EPR signal; however, TEMPO is not fully selective and may also react with superoxide-related radicals (O_2_·^−^). Compared with the untreated control experiment, the characteristic triplet signal intensity of TEMPO decreased significantly after 5 min of discharge. This quenching effect indicates that TEMPO was consumed by reactive species generated during plasma discharge, suggesting the presence of redox-active species (potentially including reducing species and superoxide-related radicals) at the gas–liquid interface. Combined with the detection of oxidative ·OH using DMPO, these observations indicate that multiple types of reactive species coexist in the microdroplet environment, which may contribute to PFAS transformation.

**Figure 6:**
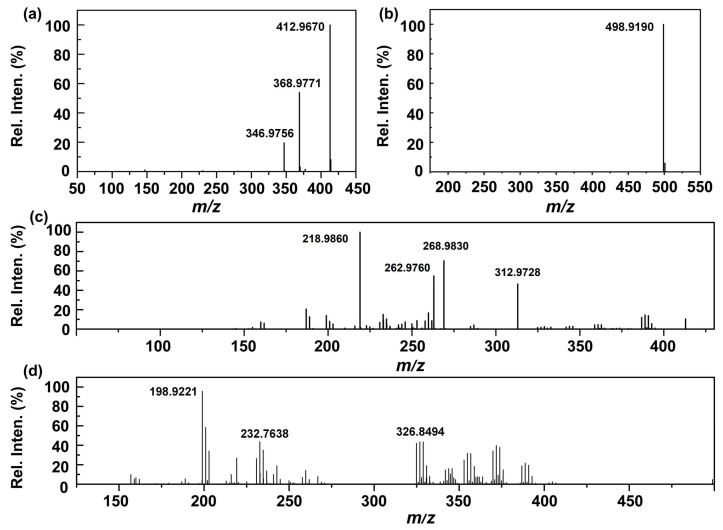
Comparison of mass spectra for PFOA and PFOS before and after plasma degradation. (**a**,**b**) Spectra of initial PFOA and PFOS, respectively; (**c**,**d**) Spectra of PFOA and PFOS after 5 min of UEN–DBD treatment.

**Figure 7:**
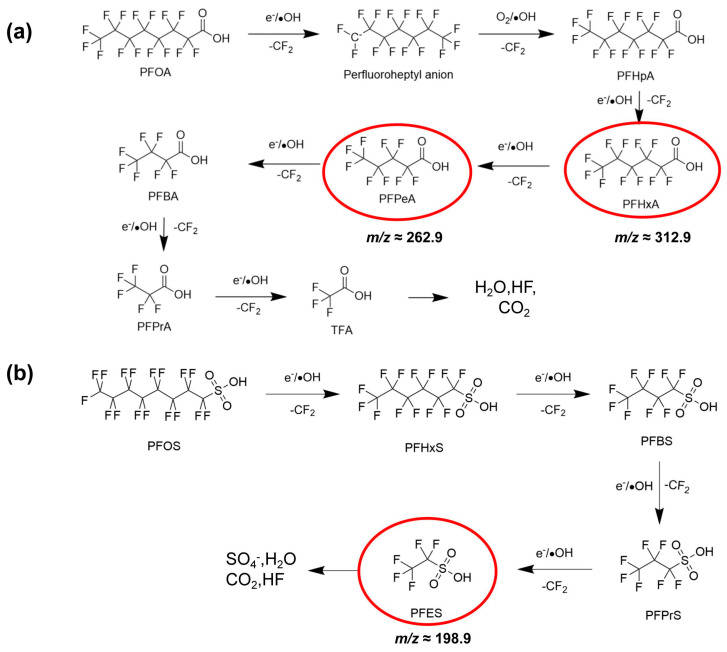
Proposed degradation pathways for (**a**) PFOA and (**b**) PFOS. The compounds highlighted by red circles represent intermediates proposed based on the mass spectrometric results.

To elucidate the degradation pathways of PFAS under UEN-DBD treatment, full-scan mass spectra was acquired using the MS detector of a Shimadzu LCMS-IT-TOF system in negative ion mode before and after plasma discharge (Figure 6). For untreated PFOA, the spectrum was characterized by a predominant peak at *m*/*z* 412.9670, corresponding to the deprotonated molecular ion [C_7_F_15_COO]^−^. Following 5 min of plasma treatment, the parent ion was strongly suppressed and several lower-*m*/*z* signals became predominant (Figure 6c). Specifically, distinct peaks observed at *m*/*z* 312.9728 and 262.9760 were tentatively assigned to the short-chain perfluorinated carboxylic acids (PFCAs): PFHxA ([C_5_F_11_COO]^−^) and PFPeA ([C_4_F_9_COO]^−^), respectively. Accompanying peaks at *m*/*z* 268.9830 and 218.9860 are consistent with the decarboxylated fluoroalkyl anions [C_5_F_11_]^−^ and [C_4_F_9_]^−^, likely resulting from the loss of a CO_2_ group (44 Da) from the corresponding intermediates. These species are consistent with characteristic fragmentation patterns observed in plasma-treated systems. A similar trend was observed for PFOS. Prior to treatment, the mass spectrum was dominated by the molecular ion of PFOS at *m*/*z* 498.9190 ([C_8_F_17_SO_3_]^−^), corresponding to the deprotonated form of PFOS. Following UEN-DBD treatment, a sharp decline in the parent ion intensity was observed, coincident with the appearance of a new major peak at *m*/*z* 198.9221. This signal is consistent with perfluoroethanesulfonate (PFES) [C_2_F_5_SO_3_]^−^, a short-chain intermediate retaining the sulfonate headgroup but containing only two –CF_2_– units. A clear shift in the dominant ions toward lower *m*/*z* values was observed for both PFOA and PFOS after UEN–DBD treatment, indicating a stepwise chain-shortening mechanism, as depicted in Figure 7. These observations suggest that the UEN–DBD system may promote C–C bond cleavage via successive removal of –CF_2_– units, consistent with the transformation of long-chain PFAS into shorter-chain products [21,22,23].

**Figure 8:**
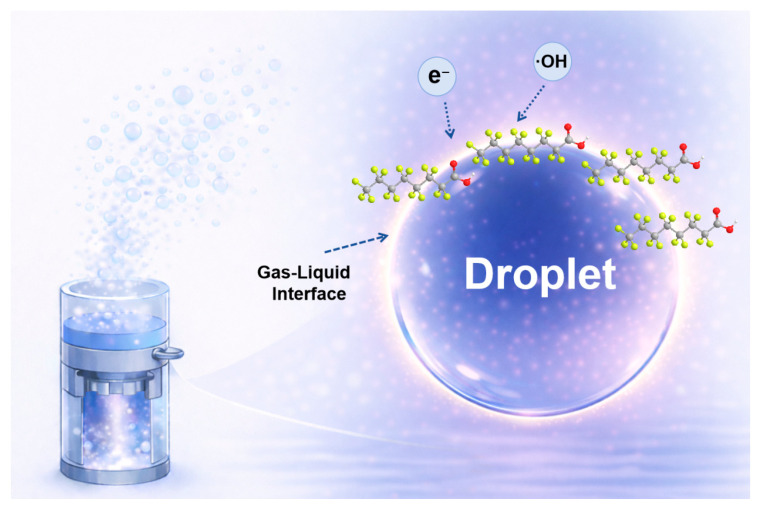
Schematic illustration of the PFAS degradation mechanism in the coupled UEN-DBD system. The diagram depicts the enrichment of PFAS molecules at the gas–liquid interface of ultrasonically generated microdroplets and their subsequent interaction with plasma-generated reactive species. In the molecular structure, gray, yellow, and red spheres represent carbon, fluorine, and oxygen atoms, respectively.

Based on the mass-spectrometric observations and supporting EPR results, the generation and interfacial transport of reactive species in the DBD plasma can be described in Figure 8. High-frequency electric field in the DBD activates gas-phase molecules (e.g., O_2_ and trace H_2_O vapor) through electron-impact processes to generate energetic electrons and reactive oxygen species such as ·OH and O_3_. The spatial behavior and specific reaction characteristics of these major species are summarized in Table S6 [24,25,26,27,28,29,30,31,32,33,34]. Under ultrasonic atomization, PFAS-containing microdroplets are continuously introduced into the discharge region, creating a highly accessible gas–liquid interface where both PFAS and short-lived reactive species are spatially concentrated. As depicted in Figure S3, the formation of this interfacial reaction layer provides a localized microenvironment that minimizes the mass-transfer distance between reactive species and the target molecules. TEMPO quenching suggests the presence of redox-active and/or reducing species at the microdroplet–plasma interface. These highly reductive species are known to participate in single-electron transfer reactions and have been widely implicated in PFAS degradation under plasma or radiolytic conditions [11]. In parallel, ·OH, directly identified by DMPO spin trapping, are generated through electron-induced dissociation of interfacial water molecules as well as secondary reactions involving dissolved ozone. Longer-lived species such as O_3_ diffuse from the gas phase into the microdroplet interior and participate in subsequent aqueous-phase reactions, contributing to the formation of superoxide (O_2_^−^) and related reactive intermediates [35,36,37,38]. Collectively, these plasma-generated species could interact with PFAS molecules or their intermediates through electron transfer, radical attack, or bond-cleavage processes, ultimately leading to C-F and/or C-S bond scission, causing progressive molecular fragmentation [39,40]. The reactions outlined above represent typical pathways reported in plasma-based systems and serve as a mechanistic framework for interpreting the mass-spectrometric evolution observed during PFAS degradation in the UEN-DBD process.

## 3. Materials and Methods

### 3.1. Experimental Setup

**Figure 9:**
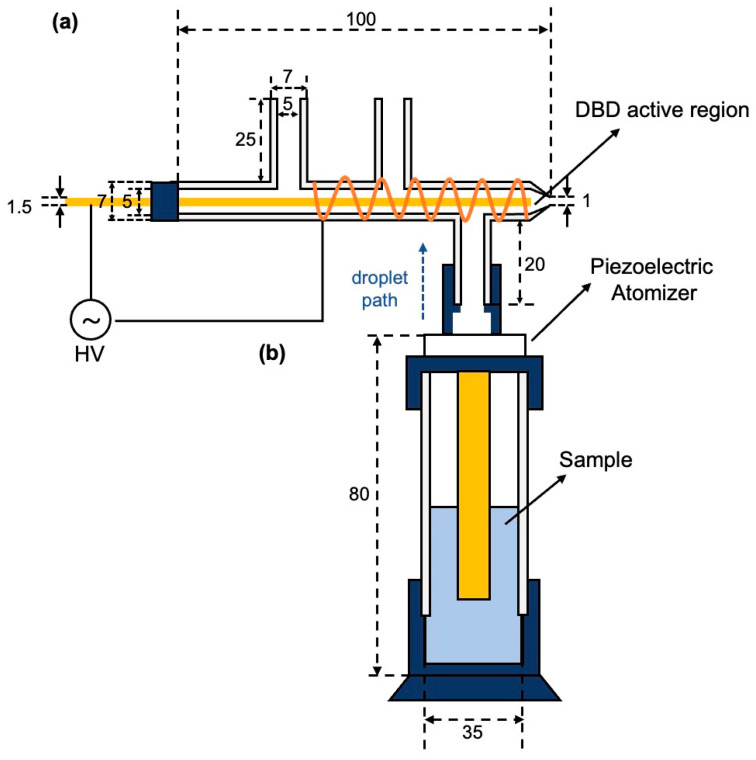
Design and assembly of the UEN–DBD system: (**a**) dielectric barrier discharge (DBD) module; (**b**) ultrasonic nebulization (UEN) module.

As illustrated in Figure 9, the UEN-DBD system consists of three main components: an ultrasonic nebulization unit (UEN), a dielectric barrier discharge module (DBD), and an electronic control/power module. The overall workflow involves liquid pollutant nebulization, in situ generation of reactive species, plasma-mediated reaction and degradation of pollutant-laden microdroplets, and condensation followed by collection of the treated effluent.

The liquid pollutant first enters the ultrasonic nebulization unit. This unit is based on a glass tube with an inner diameter of 35 mm, and a cavity is formed at the top to accommodate the ultrasonic atomizing transducer. Under ultrasonic excitation, the liquid is converted into a uniform microdroplet aerosol, which is then transported upward into the DBD reaction zone, directly entering the discharge active region. No external carrier-gas feed was supplied to the DBD tube; the atomized microdroplet aerosol was introduced into the DBD module under ambient conditions. During this process, the pollutant is enriched within the microdroplets, creating a high specific interfacial area (gas–liquid interface) that provides favorable conditions for subsequent interfacial reactions. After the discharge treatment, the microdroplet aerosol gradually deposited and coalesced on the inner wall of the DBD glass chamber, and the resulting condensate was recovered through the front tapered opening and collected into a glass vial for subsequent LC–MS/MS quantification.

The DBD module is built from a custom π-shaped quartz tube (total length: 100 mm) consisting of one main channel and three side branches. The main channel has an outer diameter of 7 mm and an inner diameter of 5 mm, and each side branch has the same dimensions. The lower side branch connects to the UEN module to introduce PFAS-containing microdroplets into the main channel, while the two upper side branches were reserved for potential auxiliary gas or sample introduction; however, no external carrier-gas feed was used in the present experiments. A 1.5 mm brass rod is inserted into the main channel as the inner electrode with an adjustable axial position, and a brass mesh is wrapped around the outer wall as the outer electrode. The inner electrode is centered in the tube, giving a radial electrode-to-wall gap of 1.75 mm (ID = 5 mm; electrode diameter = 1.5 mm). Driven by an alternating high-voltage power supply, the device generates a stable and spatially uniform discharge region (Figure 9a). In this configuration, the discharge and the subsequent plasma–microdroplet reactions occur within the internal channel (enclosed reaction zone).

### 3.2. Circuit Design

The electronic system comprises two submodules: (i) a high-voltage power supply for driving the DBD, and (ii) a low-voltage excitation circuit for actuating the ultrasonic nebulization transducer. The detailed description of the circuit design is provided in Supporting Information (SI).

#### 3.2.1. High-Voltage Driving Circuit for DBD

**Figure 10:**
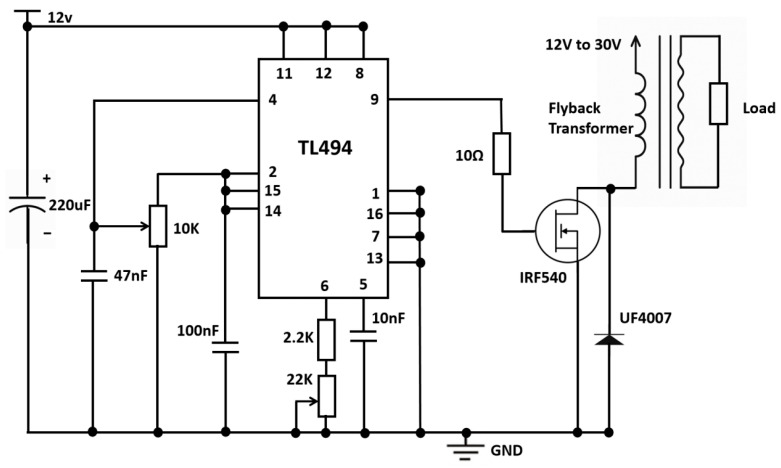
High-voltage power-supply topology for DBD. The output voltage is adjustable from 0 to 3000 V, with an effective operating frequency of approximately 38 kHz.

A flyback step-up topology based on the TL494 pulse-width modulation (PWM) controller was employed, as shown in Figure 10. Powered by a 12 V DC input, the TL494 generates high-frequency gate signals (≈38 kHz) to switch the main MOSFET (IRF540) [41], thereby driving the primary of the high-voltage transformer. The boosted secondary voltage is rectified and delivered to the discharge load, while the output level is regulated by adjusting the PWM duty ratio through the error-amplifier feedback path. In addition, an ultrafast recovery diode (UF4007) is used to suppress transient spikes and improve switching reliability. This topology offers a compact structure and stable kV-level output, meeting the requirements for safe and repeatable DBD operation under laboratory conditions.

#### 3.2.2. Excitation Circuit for Ultrasonic Nebulization

**Figure 11:**
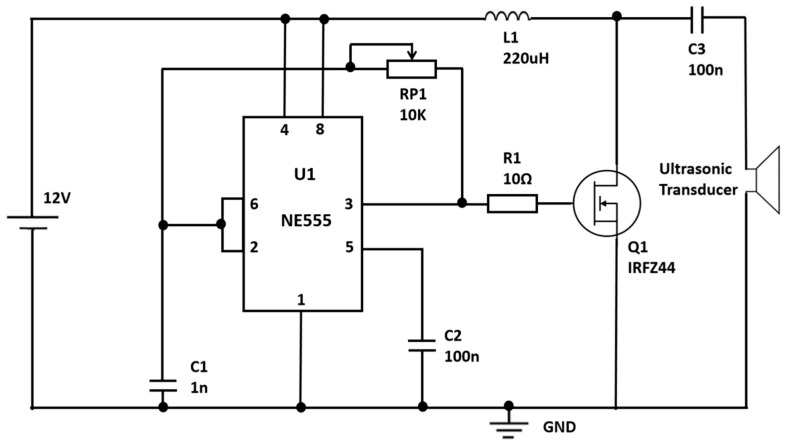
Excitation-circuit topology for driving the ultrasonic nebulization transducer.

An astable oscillator based on the NE555 timer was implemented to drive the ultrasonic transducer, as shown in Figure 11. With a 12 V supply, the timer is configured to generate a quasi-stable square-wave signal at the target operating frequency, and the excitation level can be tuned by selecting the timing components and the power stage parameters. The output signal is routed through a current-limiting/impedance-matching path to protect the driver and improve waveform stability during nebulization [42]. Owing to its simplicity and controllability, the driver can be readily adapted to different transducers or experimental media by adjusting the excitation frequency and amplitude.

### 3.3. Chemical Reagents and Samples

Two representative PFAS, PFOA and PFOS, were selected as the target contaminants. Both standards were purchased from Tianjin Alta Technology Co., Ltd. (Tianjin, China; 100 ppm stock solutions; purity ≥ 98%). The commercial 100 ppm PFAS standards were first diluted with methanol to 10 ppm intermediate solutions and then further diluted with ultrapure water (18.2 MΩ·cm) to the desired working concentrations prior to use (prepared fresh before use). All solutions were pretreated by filtration through 0.22 μm hydrophilic polyethersulfone (PES) syringe filters to remove particulate impurities and to minimize losses associated with adsorption [43,44]. Previous studies have shown that PES membranes do not produce detectable PFAS extractables and exhibit substantially lower adsorption of long-chain PFAS than polytetrafluoroethylene (PTFE), glass fiber, or polypropylene filters, making them well suited for sample pretreatment in PFAS analysis. In addition, the recovered liquid volume was kept as consistent as possible across experiments to reduce uncertainties arising from volume variations.

### 3.4. Experimental Setup and Treatment Procedures

To systematically compare the effects of ultrasonic nebulization-based enrichment, standalone dielectric barrier discharge (DBD) treatment, and the coupled UEN-DBD process on PFAS, a series of comparative and time-resolved experiments were conducted. First, aqueous solutions of PFOA and PFOS with initial concentrations of 1.0, 0.5, 0.1, and 0.01 ppm were subjected to ultrasonic nebulization at 108 kHz. After condensation, the collected liquid was analyzed by LC-MS, and enrichment factors were calculated by comparison with untreated controls. For the standalone DBD treatment, the same custom DBD reactor and electrical conditions were used, but the solution was introduced directly into the quartz DBD channel without ultrasonic nebulization (i.e., no microdroplet generation), and the treated liquid was then collected for LC–MS analysis. In the coupled UEN-DBD configuration, the solutions were continuously nebulized and delivered in real time into the DBD region, where plasma treatment was applied under the same operating conditions (2.5 kV, 5 min). Upon completion, the condensate was collected and analyzed to evaluate the synergistic degradation performance. To ensure direct comparability among different experimental conditions, the solution composition, discharge voltage, and treatment duration were kept identical across all experiments. During plasma treatment, the input voltage and current were maintained at 13.8 V and 0.5 A, corresponding to an electrical power of approximately 6.9 W. Finally, using 0.1 ppm as a representative concentration, time-resolved experiments were performed under the UEN-DBD configuration with treatment durations ranging from 1 to 5 min to elucidate the influence of reaction time on degradation efficiency. Time-dependent experiments were conducted as independent runs using freshly prepared solutions treated for the specified durations. The treatment time refers to the plasma-on (discharge) duration during which the aerosol microdroplets were delivered into the discharge active region, followed by condensation and collection for LC–MS/MS analysis. Further details on the quantitative analysis method are given in the Supporting Information. For representative PFOA/PFOS solutions (1.0 and 0.5 ppm, 5 min), fluoride ions (*F^−^*) in the collected condensate were quantified and used to calculate the defluorination degree (deF, %; see Supporting Information). All experiments were performed in triplicate (*n* = 3), and error bars in the figures represent the standard deviation (SD). Representative photographs of the experimental setup, operating states, and sample collection procedure are provided in Figure S5 in the Supporting Information for additional clarification.

### 3.5. Analytical Methods

#### 3.5.1. Quantification of PFAS

Quantitative analysis was performed using an ExionLC^TM^ AD system coupled to a QTRAP^®^ 6500+ triple quadrupole/linear ion trap mass spectrometer (AB Sciex, Framingham, MA, USA). Chromatographic separation was achieved on a COSMOSIL C18 column (2.1 mm × 100 mm, 1.8 μm) at 40 °C with a flow rate of 0.30 mL·min^−1^. The injection volume was 1.0 μL. Mobile phase A consisted of 2 mM ammonium acetate in water containing 0.01% formic acid, and mobile phase B was methanol. The gradient program was set as follows: 0.00–1.00 min, 95/5 (A/B); 1.00–5.00 min, linear ramp to 2/98; 5.00–7.00 min, held at 2/98; 7.00–7.10 min, returned to 95/5; and 7.10–9.00 min, maintained at 95/5 for column re-equilibration. The autosampler temperature was maintained at 15 °C. The aspiration/dispense rate was 5 μL·s^−1^. The needle-wash mode was set to External only, with a 3 s pre- and post-aspiration soak and an additional 2 s rinse at the rinse port.

Mass spectrometric detection was operated in negative electrospray ionization mode (ESI^−^) with an IonSpray voltage of −3.5 kV. Source parameters were as follows: ion source gas 1 (GS1), 50 psi; gas 2 (GS2), 55 psi; curtain gas (CUR), 35 psi; collision gas (CAD), 9; and source temperature, 250 °C. All gases were high-purity nitrogen (≥99.99%).

Multiple reaction monitoring (MRM) was used for quantification with the following transitions and compound-dependent parameters: PFOS: *m*/*z* 498.9 → 80.0 and 498.9 → 99.0; DP, −100 V; EP, −10 V; CE, −30 V; CXP, −10 V; dwell time, 100 ms. PFOA: *m*/*z* 412.9 → 368.9 and 412.9 → 168.9; DP, −35 V; EP, −10 V; CE, −25 V; CXP, −10 V; dwell time, 100 ms. Specific parameters and program settings are provided in Tables S2–S4.

#### 3.5.2. Qualitative Analysis of PFAS

Qualitative analysis of degradation products was performed using a Shimadzu LCMS-IT-TOF liquid chromatography–ion trap time-of-flight mass spectrometry system (Shimadzu Corporation, Kyoto, Japan), with the MS detector used for product identification. Mass spectrometric detection was operated in negative electrospray ionization mode (ESI^−^) with a spray voltage of −3.5 kV. The Heat Block/CDL temperature was set to 200 °C. Nitrogen was used as the nebulizing gas at 1.5 L·min^−1^ and as the drying gas at 100 kPa. Data acquisition was conducted in a narrow-range full-scan mode (Segment 1/Event 1). The scan range was set to *m*/*z* 50–450 for PFOA-related analyses and *m*/*z* 125–500 for PFOS-related analyses. The scan time was 300 ms with a cycle time of 0.30 s, and the ion accumulation time was set to 10.00 ms. Each sample was measured in triplicate to improve the signal-to-noise ratio and data reproducibility. Possible transformation products were inferred by comparing the full-scan mass spectra before and after plasma treatment. These products were not isolated or quantitatively determined with authentic standards; therefore, the product analysis in this work should be interpreted as qualitative and tentative.

#### 3.5.3. Fluoride Determination and Defluorination Calculation

Fluoride ions (*F*^−^) in the collected condensate were measured using a fluoride ion meter (Leici PXBJ-286F, INESA Scientific Instrument Co., Ltd., Shanghai, China) equipped with a fluoride ion-selective electrode. The instrument was calibrated prior to each measurement batch using fluoride standard solutions, and samples were measured in triplicate. The defluorination degree (deF, %) was calculated from the net fluoride release Δ[*F^−^*] = [*F^−^*]_t_ − [*F^−^*]_0_ relative to the initial total fluorine of the parent PFAS (PFOA: *n_F_* = 15; PFOS: *n_F_* = 17), as detailed in the Supporting Information.

#### 3.5.4. Reactive Species Detection Methods

EPR spectroscopy was employed to identify the dominant reactive species generated during the UEN-DBD process. The detection of ·OH was performed using a spin-trapping technique with 5,5-dimethyl-1-pyrroline N-oxide (DMPO). Prior to the UEN-DBD treatment, 200 µL of 100 mM DMPO solution was mixed with the target solution. The mixture was then subjected to the plasma discharge (2.5 kV, 5 min). Immediately after treatment, the effluent was collected and transferred to a quartz capillary for the analysis of the characteristic DMPO-OH adduct signal. To probe the presence of hydrated electrons (e_aq_^−^) and other reducing species, 2,2,6,6-tetramethylpiperidine-1-oxyl (TEMPO) was utilized as a scavenger. In a parallel experiment, 200 µL of 100 mM TEMPO was introduced into the solution before the discharge process. The consumption of e_aq_^−^ or O_2_·^−^ was qualitatively assessed by comparing the EPR spectrum of the treated sample with that of the untreated baseline solution. The attenuation of the TEMPO signal intensity provided evidence for the involvement of e_aq_^−^ and O_2_·^−^ under the present experimental conditions.

## 4. Conclusions

Plasma-assisted degradation of PFOA and PFOS was significantly promoted in the UEN-DBD system through microdroplet-mediated interfacial enrichment. Experimental observations indicate that plasma-generated reactive species, including ·OH and other redox-active species, are present at the gas–liquid interface and may interact with PFAS molecules. The localization of these reactive species at microdroplet interface enables stepwise chain-shortening reactions, as confirmed by the formation of lower-molecular-weight intermediates identified by high-resolution mass spectrometry. The high removal efficiencies achieved within 5 min highlight the critical role of interfacial accessibility in overcoming mass transfer barriers and utilizing short-lived species. These findings provide mechanistic insight into interfacial plasma-assisted PFAS transformation and may help guide the further development of plasma-based treatment strategies for persistent contaminants.

## Figures and Tables

**Table 1:** Enrichment factors of PFOA and PFOS at different initial concentrations. Enrichment factor was calculated as C_after_/C_0_, where Cafter is the PFAS concentration measured in the collected microdroplet condensate after atomization.

Compound	Initial Concentration (ppm)	Enrichment
PFOA	1.0	100.00% ± 4.36%
	0.5	133.50% ± 4.82%
	0.1	149.54% ± 5.48%
	0.01	159.78% ± 6.74%
PFOS	1.0	100.00% ± 5.20%
	0.5	121.36% ± 5.66%
	0.1	148.00% ± 6.73%
	0.01	231.45% ± 11.26%

**Table 2:** Degradation efficiencies of standalone DBD treatment and the coupled UEN-DBD process at different initial concentrations.

Compound	Initial Concentration (ppm)	Degradation (DBD)	Degradation (UEN-DBD)
PFOA	1.0	10.38% ± 2.50%	36.00% ± 5.01%
	0.5	36.23% ± 4.52%	50.85% ± 5.00%
	0.1	38.26% ± 4.02%	85.05% ± 7.03%
	0.01	85.82% ± 4.01%	96.06% ± 4.00%
PFOS	1.0	28.66% ± 2.01%	41.40% ± 2.50%
	0.5	33.00% ± 2.00%	49.09% ± 3.57%
	0.1	52.44% ± 3.15%	78.60% ± 3.50%
	0.01	73.42% ± 4.12%	94.86% ± 3.01%

**Table 3:** Calculated energy yield values (μg kJ^−1^) for PFAS removal in DBD and UEN–DBD processes under different initial concentrations.

Compound	Initial Concentration (ppm)	Energy Yield of DBD (μg kJ^−1^)	Energy Yield of UEN-DBD (μg kJ^−1^)
PFOA	1.0	2.51 ± 0.64	8.70 ± 1.21
	0.5	4.38 ± 0.55	6.14 ± 0.60
	0.1	1.24 ± 0.57	2.05 ± 0.17
	0.01	0.21 ± 0.01	0.23 ± 0.01
PFOS	1.0	6.90 ± 0.49	10.00 ± 0.61
	0.5	3.99 ± 0.24	5.93 ± 0.43
	0.1	1.27 ± 0.08	1.90 ± 0.08
	0.01	0.18 ± 0.01	0.23 ± 0.01

**Table 4:** Fluoride concentration (*F^−^*) and defluorination (deF, %) for PFOA and PFOS after 5 min treatment at 1.0 and 0.5 ppm.

Compound	Initial Concentration (ppm)	Process	Fluoride Concentration After Treatment, $F^{-}\;(\mu g/L)$	Defluorination (deF, %)
PFOA	1.0	DBD	33.1 ± 4.5	4.8 ± 0.7
		UEN-DBD	101.3 ± 6.5	14.7 ± 0.9
	0.5	DBD	29.3 ± 2.3	8.5 ± 0.7
		UEN-DBD	62.0 ± 2.9	18.0 ± 0.9
PFOS	1.0	DBD	48.5 ± 5.1	7.5 ± 0.8
		UEN-DBD	102.1 ± 5.6	15.8 ± 0.9
	0.5	DBD	22.6 ± 2.3	7.0 ± 0.7
		UEN-DBD	54.9 ± 2.7	17.0 ± 0.8

**Table 5:** Residual concentrations and degradation efficiencies as a function of treatment time under the coupled UEN-DBD process.

Treatment Time (min)	PFOA Residual Concentration in the Original Sample (ppb)	Degradation (PFOA)	PFOS Residual Concentration in the Original Sample (ppb)	Degradation (PFOS)
1	67.6	32.40%	85.2	14.80%
2	61.4	38.60%	60.2	39.80%
3	48.0	52.00%	45.8	54.20%
4	30.0	70.00%	24.2	75.80%
5	20.0	80.00%	21.4	78.60%

## Data Availability

The data supporting the findings of this study are available within the article and its Supporting Information. Additional data are available from the corresponding author upon reasonable request.

## Supplementary Materials

The following supporting information can be downloaded at https://www.mdpi.com/article/10.3390/molecules31071157/s1, Figure S1: PFOA calibration curve; Table S1: Calibration-point data for PFOA; Figure S2: LC–MS results for a 1 ppm PFOA sample under different treatment conditions (all samples were diluted 50-fold prior to analysis). From top to bottom: standard solution, ultrasonically nebulized sample, DBD-treated sample, and UEN–DBD-treated sample; Table S2: Gradient elution program for quantitative LC–MS/MS analysis of PFOA and PFOS (Mobile phases: A, 2 mM ammonium acetate in water containing 0.01% formic acid; B, methanol.); Figure S3: Schematic diagram of the distribution and action of active species at the gas–liquid interface; Table S3: ESI source parameters (negative ion mode) for quantitative LC–MS/MS analysis; Figure S4: Representative steady-state waveforms of the applied voltage and current in the DBD reactor. (reported as Vrms ≈ 2.5 kV); Table S4: MRM transitions and compound-dependent parameters for quantitative determination of PFOA and PFOS; Figure S5: Representative photographs of the UEN–DBD operating and condensate collection process: (a) standalone UEN showing stable mist generation; (b) UEN connected to the DBD quartz tube, showing droplet introduction into the tube; (c) UEN–DBD operation showing mist inside the tube during plasma discharge; (d) collection of the condensate by tilting the DBD tube after treatment; Table S5: Quantification results for 1 ppm PFOA under different treatment conditions (all samples were diluted 50-fold prior to LC–MS analysis); Table S6: Reaction characteristics and spatial behavior of major reactive species in the UEN–DBD system.

## Abbreviations

The following abbreviations are used in this manuscript:

PFAS	Per- and polyfluoroalkyl substances
PFOA	perfluorooctanoic acid
PFOS	perfluorooctanesulfonate
PFCAs	short-chain perfluorinated carboxylic acids
PFES	perfluoroethanesulfonate
PFHxA	Perfluorohexanoic acid
PFPeA	Perfluoropentanoic acid
POPs	persistent organic pollutants
PES	polyethersulfone
PTFE	Polytetrafluoroethylene
TEMPO	2,2,6,6-tetramethylpiperidine-1-oxyl
DMPO	5,5-dimethyl-1-pyrroline N-oxide
AOPs	Advanced oxidation processes
·OH	hydroxyl radicals
e_aq_^−^	hydrated electrons
LC	Liquid Chromatography
MS	mass spectrometry
LC-MS	liquid chromatography–mass spectrometry
UEN	ultrasonic nebulization unit
DBD	Dielectric barrier discharge
UEN-DBD	ultrasonic atomization–dielectric barrier discharge
EPR	electron paramagnetic resonance
PWM	pulse-width modulation
MRM	Multiple reaction monitoring
SI	Supporting Information
