# Supplementary material for: Enhanced Interfacial Plasma Degradation of Per- and Polyfluoroalkyl Substances (PFAS) via Ultrasonically Generated Microdroplets
Source: Molecules. 2026 Mar 31;31(7):1157. doi: 10.3390/molecules31071157 (PMC13075150; doi:10.3390/molecules31071157)
Supplement: Supplementary file 1 [file molecules-31-01157-s001.zip › molecules-4174727-supplementary.pdf]

Enhanced Interfacial Plasma Degradation of Per- and Polyfluoroalkyl Substances (PFAS) via Ultrasonically Generated Microdroplets

Ao Chen, Haoyu Yuan, Zhengtong Qiu and Chaonan Mu*

State Key Laboratory of Marine Resource Utilization in South China Sea, School of Marine Sciences, Hainan University, Haikou 570228, China

*Corresponding author:

E-mail address: chaonanmu@hainanu.edu.cn (C. Mu).

1. Analytical Methods and Quantification Procedures

1.1 External Calibration and Sample Preparation

Per- and polyfluoroalkyl substances (PFASs) were quantified using an external calibration approach. Seven calibration levels were prepared by serial dilution of PFOA and PFOS stock solutions. Calibration curves were obtained by linear regression of peak area versus concentration.Samples were diluted as needed to keep signals within the linear range.

A dilution factor (*DF*) was selected based on the expected concentration.

Peak areas were converted to the vial concentration (C_{inj}) using the calibration curve.. The concentration in the treated solution was back-calculated as:

$$C_{sample} = C_{inj} * DF \tag{S1}$$

1.2 Performance metrics.

To compare the performance of ultrasonic nebulization (UEN), standalone dielectric barrier discharge (DBD), and the coupled UEN–DBD system, degradation efficiency, enrichment factor, and energy yield were calculated. For each analytical batch and nominal initial concentration, the untreated solution was used as the reference concentration (C_0). Concentrations after treatment were denoted as $C_{\mathrm{after.}}$

Degradation efficiency (η,%):

$$\eta(\%) = \frac{C_0 - C_{after}}{C_0} * 100\% \tag{S2}$$

Enrichment factor *(EF*,%):

$$EF(\%) = \frac{C_{after}}{C_0} * 100\% \tag{S3}$$

Energy yield (μg kJ^{-1}):

Energy yield (*Y*) was defined as the mass of PFAS removed per unit electrical energy input:

$$Y = \frac{(C_0 - C_{after})V}{PT} = \frac{C_0 \eta V}{PT} \tag{S4}$$

where C_0 and C_{after} are the PFAS concentrations before and after treatment, *V* is the treated volume, *P* is the input power, *T* is the treatment time, and η is the degradation efficiency. Concentrations and volume were converted to yield m_{removed} in μg, and *PT* was converted to kJ.

Notes on energy accounting and interpretation. The energy yield reported in this work is calculated based on the removal of the parent PFAS (PFOA/PFOS) quantified by LC–MS/MS, and therefore reflects parent-compound removal/transformation rather than complete mineralization (e.g., TOC removal or a full fluorine mass balance). The input power P was estimated from the DC input to the power module, which was maintained at approximately 13.8 V and 0.5 A during operation (i.e., ~6.9 W; minor fluctuations may occur). The electrical energy consumption was calculated as E=P×T. For the UEN–DBD configuration, the electrical consumption of the ultrasonic nebulization unit was not included in the present energy-yield calculation; thus, the reported value should be interpreted as a plasma-side (boundary-limited) energy yield. We note that this energy yield is most suitable for internal comparisons within this study, and should not be directly compared to absolute system-level energy efficiencies reported in other studies.

Defluorination (Def,%):

The fluoride ion concentration measured in the collected condensate is denoted as $[F^{-}]t$, while the background fluoride concentration in the untreated control (blank) is denoted as $[F^{-}]_0$. The net fluoride release is defined as:

$$\Delta F^{-} = [F^{-}]_t - [F^{-}]_0 \tag{S5}$$

The defluorination degree (deF, %) was calculated based on fluoride release relative to the initial total fluorine of the parent PFAS:

$$deF(\%) = \frac{\Delta[F^{-}]}{n_F . C_0} \times 100\% \tag{S6}$$

where C_0 is the initial concentration of the parent PFAS (mol·L^{-1}) and n_F is the number of fluorine atoms in the parent molecule (PFOA: n_F=15; PFOS:n_F=17).

1.3 Quantitative method example (PFOA at 1 ppm concentration)

The PFOA solution （1 ppm) was used to demonstrate the quantification procedure. Figure S2 shows the extracted ion chromatograms (EICs) for the standard and for samples treated by UEN, DBD,

and UEN–DBD (Samples were diluted 50-fold prior to LC–MS analysis to minimize potential matrix effects and ensure the detector response within the linear dynamic range.). A distinct peak was observed at ~8.3 min across all conditions, with stable retention time.LC–MS/MS conditions and MRM parameters are provided in Tables S2–S4.

Seven calibration levels were used to build the PFOA calibration curve (Figure S1). Calibration-point peak areas and back-calculated concentrations are listed in Table S1. The resulting calibration curve was then applied to back-calculate PFOA concentrations for the treated samples, and the quantification results are summarized in Table S5.

2.Experimental Setup and Operating Procedure

During operation, the ultrasonic nebulizer generated a stable visible mist, and the resulting droplets were introduced into the DBD quartz tube. Under simultaneous UEN–DBD operation, mist/droplets were present inside the tube while plasma discharge was maintained. After treatment, the droplets naturally condensed on the inner wall of the DBD glass tube, and the condensate was collected by gently tilting the tube and pouring the liquid out through the conical opening at the front end for subsequent analysis. Representative photographs of the operating and condensate collection process are shown in Figure S5.

3.Degradation Kinetic Analysis of PFOA and PFOS

As shown in Figure 4 (main text), the normalized concentrations (C_t/C_0) of PFOA and PFOS decreased with treatment time. The data were fitted with a pseudo-first-order model:

$$\frac{C_t}{C_0} = e^{-kt} \tag{S5}$$

where t is the treatment time (min). The fitted relationships were $C_t/C_0=e^{-0.288t}$ for PFOA and $C_t/C_0=e^{-0.307t}$ for PFOS. Accordingly, k values were 0.288 min^{-1} ($R^2=0.9528$) for PFOA and 0.307 min^{-1} ($R^2=0.9564$) for PFOS.

4. High-voltage driving circuit for DBD

A compact flyback step-up driver was implemented to supply kV-level excitation for the DBD reactor from a 12 V DC input. PWM gate pulses were generated by a TL494 controller (single-ended configuration; output at pin 9) and applied to an IRF540 power MOSFET through a 10 Ω gate resistor. The MOSFET switched the primary of a high-voltage transformer with a turns ratio of 150:1, and the secondary output was rectified before being applied to the discharge load.

During operation, the switching frequency was continuously adjustable within 30–45 kHz via a sliding potentiometer in the TL494 timing network (pins 5–6). The output level was controlled by tuning the PWM duty ratio through the TL494 error-amplifier control path, enabling an adjustable discharge voltage of 0–3 kVpp under the experimental conditions used in this work. An ultrafast recovery diode (UF4007) was incorporated in the power stage to improve switching robustness and suppress transient over-voltage during commutation.

5.Excitation circuit for ultrasonic nebulization

The ultrasonic transducer was actuated by a 12 V DC excitation circuit based on an NE555 timer configured in astable mode. The square-wave output (pin 3) was used to drive an IRFZ44 MOSFET through a 10 Ω gate resistor (R1). The MOSFET output stage was connected in series with an inductor (L1 = 220 μH) and the ultrasonic transducer, and a decoupling capacitor (C3 = 100 nF) was placed near the power stage to maintain stable excitation.

The excitation frequency was set by the NE555 timing network (RP1, 10 kΩ, and C1, 1nF) and was tuned to 108 kHz for the nebulization experiments reported herein.

Table S1. Calibration-point data for PFOA.

Standard concentration (ppb)	Peak area	Back-calculated concentration (ppb)	Relative accuracy (%)
0.5	225500	0.549	+9.8
1	341700	0.896	−10.4
2	713800	2.01	+0.5
5	1830000	5.34	+6.8
10	3489000	10.3	+3.0
20	5812000	17.2	−14.0
50	17510000	52.2	+4.4

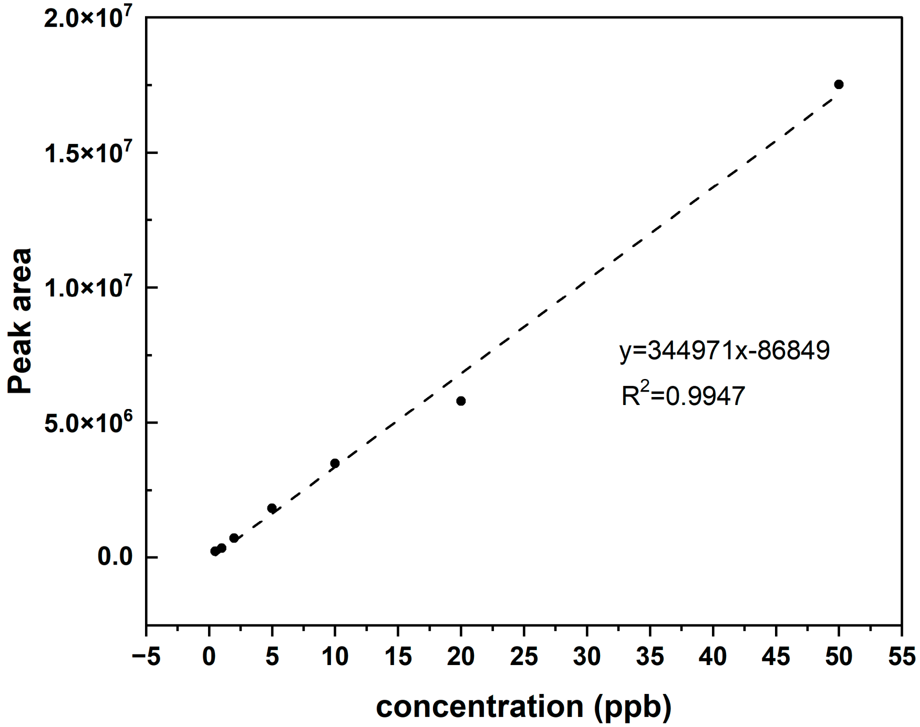

Figure S1. PFOA calibration curve.

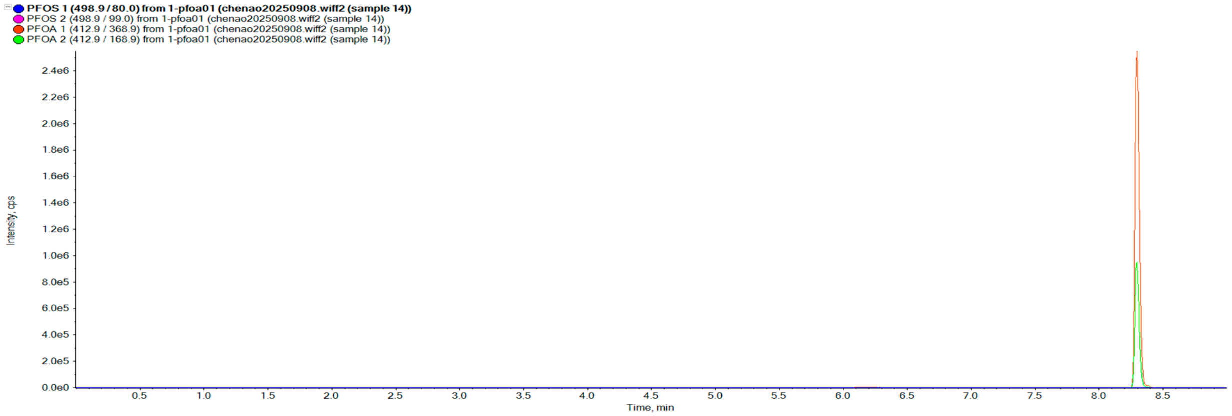
PFOS 1 (498.9 / 80.0) from 1-pfoa01 (chenao20250908.wiff2 (sample 14))
PFOS 2 (498.9 / 99.0) from 1-pfoa01 (chenao20250908.wiff2 (sample 14))
PFOA 1 (412.9 / 368.9) from 1-pfoa01 (chenao20250908.wiff2 (sample 14))
PFOA 2 (412.9 / 168.9) from 1-pfoa01 (chenao20250908.wiff2 (sample 14))
Intensity, cps
Time, min

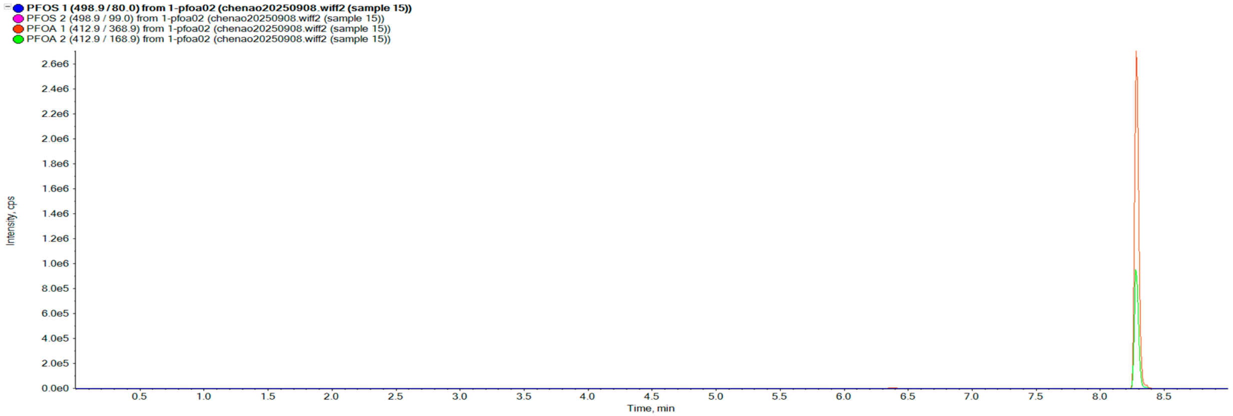
PFOS 1 (498.9 / 80.0) from 1-pfoa02 (chenao20250908.wiff2 (sample 15))
PFOS 2 (498.9 / 99.0) from 1-pfoa02 (chenao20250908.wiff2 (sample 15))
PFOA 1 (412.9 / 368.9) from 1-pfoa02 (chenao20250908.wiff2 (sample 15))
PFOA 2 (412.9 / 168.9) from 1-pfoa02 (chenao20250908.wiff2 (sample 15))
Intensity, cps
Time, min

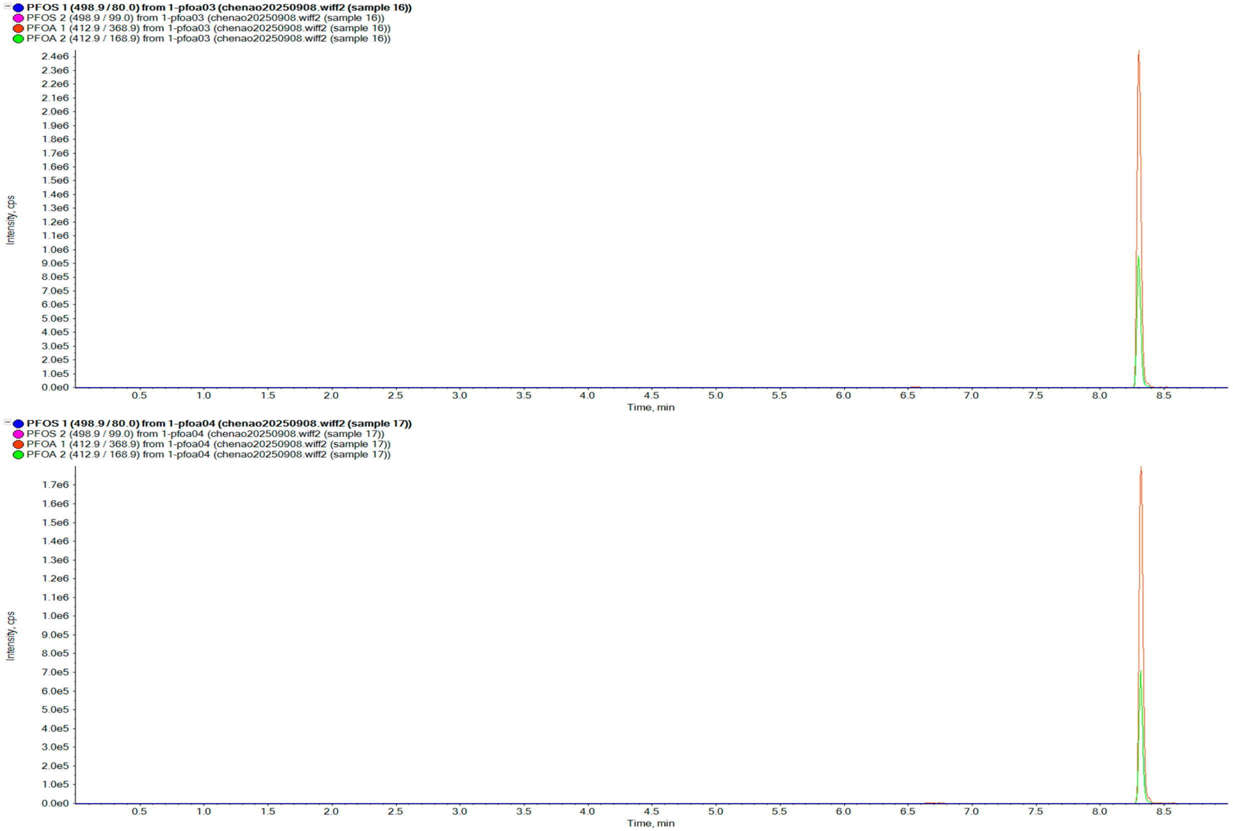

Figure S2. LC–MS results for a 1 ppm PFOA sample under different treatment conditions (all samples were diluted 50-fold prior to analysis). From top to bottom: standard solution, ultrasonically nebulized sample, DBD-treated sample, and UEN–DBD-treated sample.

Table S2 Gradient elution program for quantitative LC–MS/MS analysis of PFOA and PFOS (Mobile phases: A, 2 mM ammonium acetate in water containing 0.01% formic acid; B, methanol.).

Time (min)	%A	%B	Step
0.00–1.00	95	5	Hold
1.00–5.00	95 → 2	5 → 98	Linear ramp
5.00–7.00	2	98	Hold
7.00–7.10	2 → 95	98 → 5	Return
7.10–9.00	95	5	Re-equilibration

Table S3 ESI source parameters (negative ion mode) for quantitative LC–MS/MS analysis.

Parameter	Setting
IonSpray voltage (kV)	−3.5
Ion source gas 1 (GS1, psi)	50
Ion source gas 2 (GS2, psi)	55
Curtain gas (CUR, psi)	35
Collision gas (CAD, a.u.)	9
Source temperature (°C)	250
Gas type	High-purity nitrogen (≥99.99%)

Table S4 MRM transitions and compound-dependent parameters for quantitative determination of PFOA and PFOS.

Analyte	Precursor ion (m/z)	Product ion (m/z)	DP (V)	EP (V)	CE (V)	CXP (V)	Dwell time (ms）
PFOS	498.9	80.0	-100	-10	-30	-10	100
PFOS	498.9	99.0	-100	-10	-30	-10	100
PFOA	412.9	368.9	-35	-10	-25	-10	100
PFOA	412.9	168.9	-35	-10	-25	-10	100

Table S5. Quantification results for 1 ppm PFOA under different treatment conditions (all samples were diluted 50-fold prior to LC–MS analysis).

Sample	Peak area	Retention time (min)	Measured concentration (from calibration curve)	Actual Concentration in Sample (ppb)	Degradation / enrichment
1.Untreated solution	6160 000	8.30	18.3	915	
2.Ultrasonic nebulization	6113 000	8.28	18.1	905	No significant change

3.DBD	5517000	8.30	16.4	820	10.38%
4.UEN-DBD	3959000	8.32	11.7	585	36%

Table S6. Reaction characteristics and spatial behavior of major reactive species in the UEN–DBD system [24-34].

Reactive species	Typical rate constant (k)	Half-life	Predominant reaction zone	Primary role / mechanism
·OH	~1×10^{8} $M^{-1}\cdot s^{-1}$ (aqueous);~2.2×10^{-10} cm^3/s (gas)	<1 μs (gas)	gas–liquid interface	A strong oxidant that can attack C–F or C–S bonds and trigger chain scission.
O (atomic oxygen)	~1×10^{-10} cm^3/s (gas)	50–200 μs (gas)	discharge region and interface	Precursor to O_3 and ·OH; participates in oxidative pathways.
O_3 (ozone)	~5.5×10^{-34} cm^6/s	tens of seconds to minutes (gas)	Gas phase; dissolves into droplets	A relatively stable oxidant that can react directly or generate ·OH via reactions in water.
O_2^{-} (superoxide)	~1.0×10^{-12} cm^3/s (gas)	<1 μs (gas)	gas-phase boundary layer	Converts to HO_2· upon protonation and can contribute to oxidative chemistry.
HO_2·(hydroperoxyl radical)	10^{5}–10^{7} $M^{-1}\cdot s^{-1}$ (aqueous)	~1 ms (aqueous)	outer droplet layer / boundary	A secondary oxidant involved in chain-propagating oxidation reactions.
e_{aq}^{-}(hydrated electron)	~7.3×10^{7} M^{-1} s^{-1}(PFOS) ~5.1×10^{7} M^{-1} s^{-1}(PFOA)	ps–μs (microdischarge zone)	discharge region and droplet vicinity	A strong reductant that can induce bond cleavage and initiate radical formation.

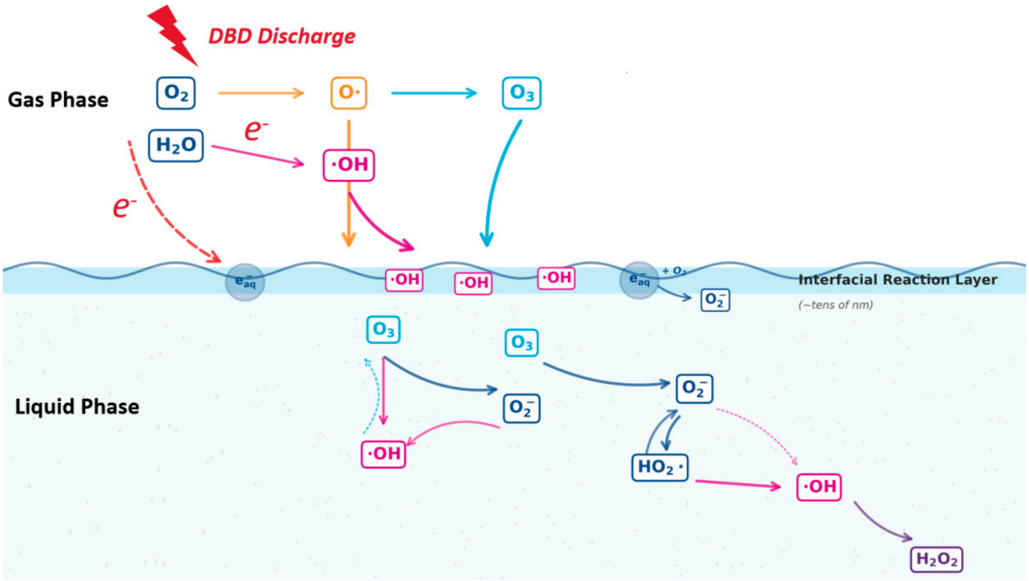

Figure S3. Schematic diagram of the distribution and action of active species at the gas-liquid interface.

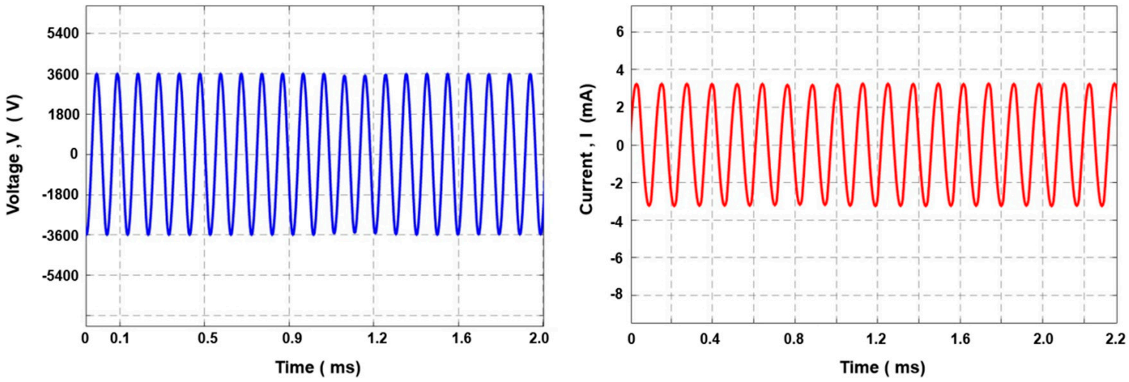

Figure S4. Representative steady-state waveforms of the applied voltage and current in the DBD reactor.(reported as Vrms ≈ 2.5 kV).

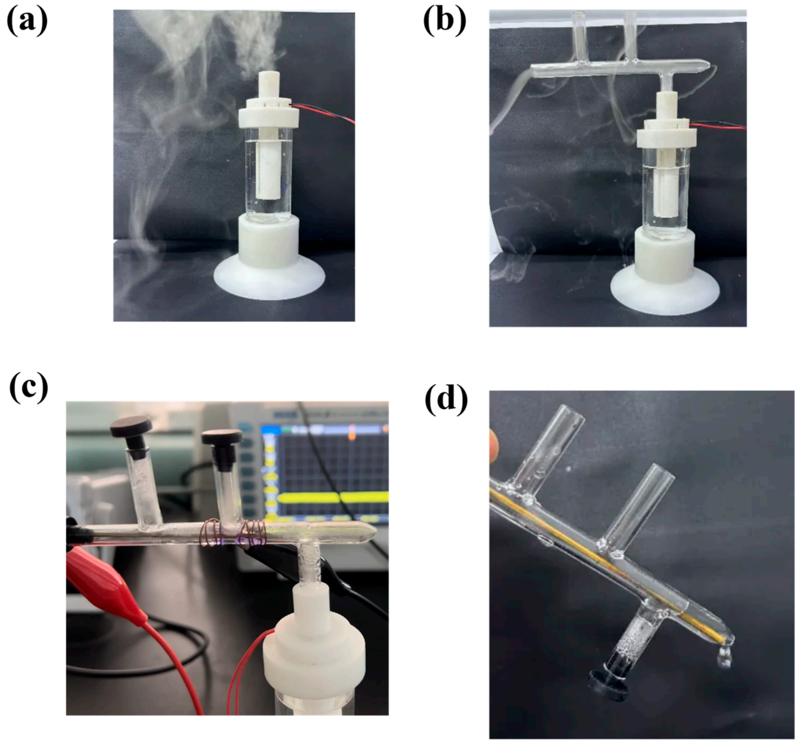

Figure S5. Representative photographs of the UEN–DBD operating and condensate collection process: (a) standalone UEN showing stable mist generation; (b) UEN connected to the DBD quartz tube, showing droplet introduction into the tube; (c) UEN–DBD operation showing mist inside the tube during plasma discharge; (d) collection of the condensate by tilting the DBD tube after treatment.
